# Renin-secreting chromophobe renal cell carcinoma: An uncommon cause of secondary hypertension in a young female

**DOI:** 10.1016/j.eucr.2025.103011

**Published:** 2025-03-15

**Authors:** Tareq Jarrar, Younes Jafar Hamam, Hadeel Abdallah Al Kayed, Bassel Esmat Alrabadi, Ayham Kamel Milhem, Maryam Hisham Alqatawna, Husam Odaibat

**Affiliations:** a Al-Quds University, Jerusalem, Palestine; b University of Jordan, Amman, Jordan; c Al Bashir Hospital, Amman, Jordan; d Jordan University of Science and Technology, Irbid, Jordan; e Yarmouk University, Irbid, Jordan; f Jordanian Royal Medical Services, Amman, Jordan

**Keywords:** Renal cell carcinoma (RCC), Chromophobe RCC, Secondary hypertension, Renin-secreting tumor, Paraneoplastic syndrome, Uncontrolled hypertension

## Abstract

Renal cell carcinoma (RCC) is a rare but significant cause of secondary hypertension. Chromophobe RCC represents 5 % of all RCC cases, with renin secretion being even rarer. We report a case of a 32-year-old female with six-month history of uncontrolled hypertension. Laboratory findings showed elevated plasma renin and aldosterone levels. Imaging revealed a large left renal mass. After radical nephrectomy, histopathology confirmed chromophobe RCC. Her blood pressure normalized postoperatively, and she was normotensive at 1-month follow-up. This case underscores the importance of considering renal tumors in hypertensive patients and the role of surgery in achieving favorable outcomes.

## 1 Introduction

**Fig. 1:**
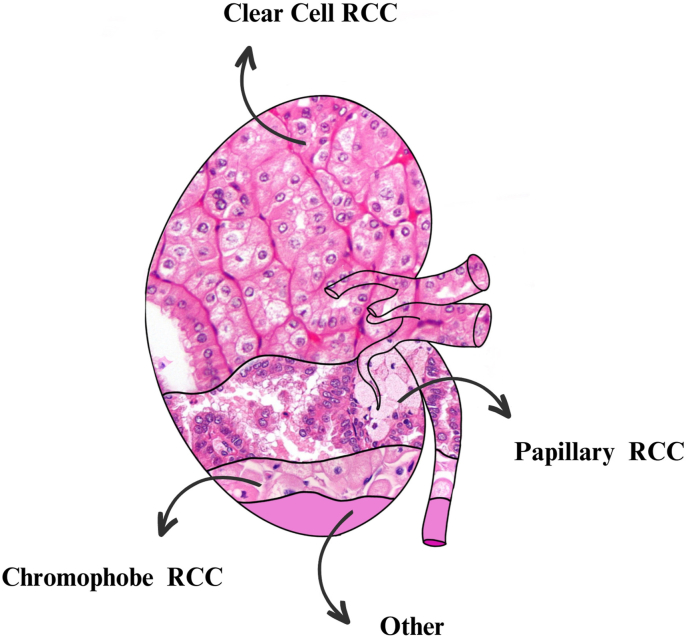
Schematic representation of the most prevalent subtypes of renal cell carcinoma (RCC).

Renal cell carcinoma (RCC) accounts for approximately 2–3 % of all adult malignancies and is the most prevalent solid kidney tumor.^1^ Among RCC subtypes, clear cell, papillary, and chromophobe RCC represent roughly 80 %, 10 %, and 5 % of cases, respectively (Fig. 1).^2^ While RCC is a rare cause of secondary hypertension, it is an important consideration in cases where hypertension is not primarily vascular in origin. Paraneoplastic events, such as those seen in translocation RCCs involving microphthalmia-associated transcription factor E3, can occasionally result in hypertension, though these are exceedingly rare.^3^

Renin-producing tumors, such as renin-secreting RCCs and juxtaglomerular cell tumors (JXG), can autonomously secrete renin, leading to activation of the systemic renin-angiotensin-aldosterone system (RAAS) and subsequent hypertension.^4^^,^^5^ Additionally, RCC may cause hypertension through a mass effect, compromising renal perfusion pressure and indirectly activating the0 RAAS.^6^^,^^7^ In rare cases, tumors containing adrenal tissue may produce renin, aldosterone, or sex steroids, contributing to hypertension or adrenogenital syndromes.^8^

Given these diverse mechanisms, renal tumors should be included in the differential diagnosis of secondary hypertension, particularly in young adults. This case report describes a 32-year-old female presenting with poorly controlled hypertension as the initial manifestation of chromophobe RCC, a rare subtype of RCC.

## 2 Case presentation

A 32-year-old female patient with an unremarkable past medical and surgical history presented to our hospital with symptoms of intermittent headache, fatigue, and poorly controlled hypertension persisting for six months. Despite adherence to multiple antihypertensive medications, including enalapril, amlodipine, and propranolol, her blood pressure remained elevated, with systolic readings ranging from 130 to 160 mmHg.

She reported no history of weight loss, cough, abdominal discomfort, skin changes, flushing, sweating, visual disturbances, or neurological symptoms. Additionally, there was no significant drug or family history of hypertension or endocrine disorders.

At presentation, her blood pressure was 130/90 mmHg. Physical examination revealed normal heart sounds without murmurs or additional sounds. Abdominal examination showed no palpable masses, tenderness, or bruits. There were no features suggestive of Cushing's syndrome, and the remainder of the physical examination was unremarkable.

Initial laboratory investigations included a complete blood count (CBC), lipid profile, hemoglobin A1c, iron studies, prolactin, and thyroid-stimulating hormone (TSH), all of which were within normal ranges. Serum cortisol levels maintained their physiological circadian rhythm. Serum electrolytes, including potassium, were within normal limits. To rule out pheochromocytoma, urinary metanephrines were obtained and found to be within normal limits. However, plasma renin activity and aldosterone levels were significantly elevated at 11.7 ng/mL/hr (reference: 0.06–4.69 ng/mL/hr) and 37.9 ng/dL (reference: 1.46–17.4 ng/dL), respectively. The renin/aldosterone ratio was 3.24 (normal: 20–40), indicative of autonomous renin secretion.

**Fig. 2:**
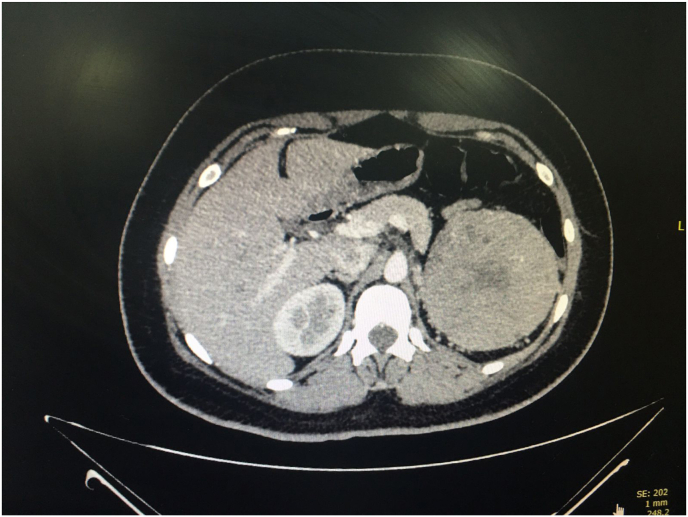
Contrast-enhanced computed tomography (CT) scan demonstrating a well-defined, mildly enhancing mass measuring 93 × 92 × 96 mm located in the upper pole of the left kidney (red arrow).

A renal ultrasound was performed, revealing a renal mass. Subsequent contrast-enhanced computed tomography (CT) identified a large, mildly enhancing mass with a central scar located in the upper pole of the left kidney (Fig. 2). The mass measured 93 × 92 × 96 mm, with its inferior portion extending into the upper calyceal group of the left kidney. No evidence of local invasion was observed on imaging. Given the clinical presentation and imaging characteristics, a renin-secreting renal tumor was suspected.

**Fig. 3:**
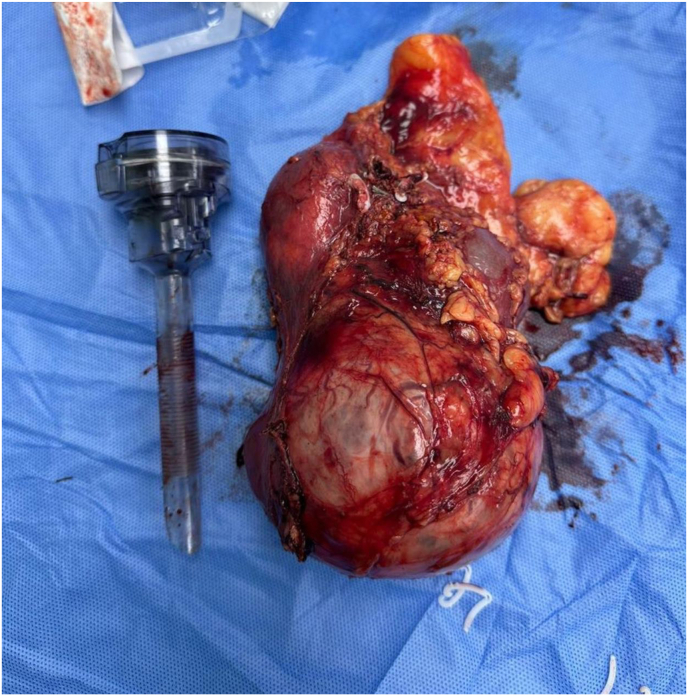
A gross image of the resected specimen showed a large mass measuring about 9 × 9 × 9 cm.

The patient underwent a left radical nephrectomy, which was successfully performed. Gross examination of the resected specimen measured 15 × 9 × 9 cm and revealed a large tan-yellow mass at the upper pole measuring 9 × 9 × 9 cm, with the tumor extending to the renal sinus (Fig. 3).

Microscopically, histopathological analysis showed large polygonal cells with perinuclear halos and prominent cell membranes, consistent with chromophobe-type histological features. Immunohistochemical staining was positive for c-kit (CD117) and cytokeratin 7 (CK7), confirming the diagnosis of chromophobe renal cell carcinoma (chRCC).

Postoperatively, the patient's blood pressure improved significantly, returning to baseline levels within one month after surgery, allowing for the reduction and eventual discontinuation of multiple antihypertensive medications.

## 3 Discussion

### 3.1 Epidemiology of chromophobe renal cell carcinoma (chRCC)

This case report describes a rare presentation of secondary hypertension caused by a renin-secreting chRCC, a tumor originating from the cells lining the distal tubules of the nephron. ChRCC is the third most common subtype of RCC after clear cell RCC (ccRCC) and papillary RCC, accounting for approximately 5 % of all RCC cases.^9^ In 2020, around 3700 new cases were reported in the United States.^10^ The mean age at diagnosis is typically in the fifth decade of life, with a reported range of 27–86 years and a slight female predominance. Most cases are diagnosed at early stages (stage I or II), while metastatic disease occurs in approximately 6 % of cases, most commonly involving the liver and lungs.^1^

### 3.2 Clinical presentation and diagnosis

The clinical course of chRCC is generally less aggressive compared to ccRCC. The classic triad of flank pain, hematuria, and a palpable abdominal mass is rare, as most patients remain asymptomatic, with renal masses often discovered incidentally during imaging performed for unrelated reasons.^10^ However, our patient presented with persistent fatigue, headache, and poorly controlled hypertension, an atypical presentation for chRCC. Nonetheless, literature suggests that fatigue occurs in 20–33 % of cases.^11^

The patient's resistant hypertension despite conventional antihypertensive therapy raised suspicion for a secondary cause of hypertension. Common differential diagnoses for secondary hypertension include endocrine disorders, such as primary hyperaldosteronism and pheochromocytoma, aortic coarctation, and renal parenchymal or vascular disease.^12^

In this case, elevated plasma renin activity and aldosterone levels, combined with ultrasound findings of a renal mass, strongly suggested a renin-secreting neoplasm. Renin-secreting tumors are categorized into three types: tumors arising from the kidney's juxtaglomerular apparatus, renin-secreting renal tumors, and extrarenal tumors such as granulosa cell tumors, lung cancer, and pancreatic cancer. The hallmark clinical features of renin-secreting tumors include hypertension, hypokalemia, and elevated plasma renin activity.

It is hypothesized that renin secretion in RCC may arise from two mechanisms. In some cases, the tumor cells themselves may autonomously produce renin 13. Alternatively, renin secretion may occur indirectly due to compression or stimulation of adjacent renal tissue, triggering activation of the RAAS. This RAAS activation contributes to the elevation of blood pressure, as observed in this case.

### 3.3 Genetic considerations in renal cell carcinoma

The National Comprehensive Cancer Network (NCCN) guidelines recommend genetic risk assessment in patients with RCC, particularly in younger adults, as hereditary factors may contribute to disease development. Genetic testing, performed via blood or saliva samples, detects germline mutations that may indicate an increased risk of malignancy.^14^ Although our patient had no significant family history or clinical features suggestive of a hereditary predisposition, genetic evaluation remains an important consideration in young individuals diagnosed with RCC. While genetic testing was not conducted in this case, its role in risk stratification, surveillance, and potential familial screening should be acknowledged.

### 3.4 Imaging and tumor characteristics

CT imaging is the preferred modality for evaluating renal masses, as it provides detailed information about both the tumor and the surrounding structures. The dynamic pattern of contrast enhancement observed on CT can assist in differentiating various subtypes of RCC.^1^

In this case, the tumor was notably larger than the mean sizes reported in the literature, which typically range from 32 mm to 70 mm in diameter. The presence of a central scar observed in the tumor is consistent with previous findings, where such a feature has been described in 19 %–34 % of chRCC cases.

The mild enhancement observed on imaging aligns with the known characteristics of chRCC, as it is a moderately vascular tumor that enhances less than the renal cortex across all phases of contrast imaging.^11^

### 3.5 Histopathological findings

chRCC is characterized histologically by large polygonal cells with prominent cell membranes, pale eosinophilic cytoplasm, and perinuclear halos, all of which were present in the tumor resected from this patient.^11^ The observed involvement of the renal sinus on histopathological examination emphasizes the importance of early surgical intervention to prevent local invasion and potential metastasis.

Differentiating chRCC from renal oncocytoma can be challenging despite distinct histopathological and immunohistochemical features. Macroscopically, chRCC typically presents with a well-circumscribed cut surface ranging from light tan to brown, whereas oncocytoma exhibits a cut surface with hues ranging from mahogany brown to tan or yellow. Immunohistochemical staining aids in their distinction, as chRCC is generally positive for CD117 and CK7, while oncocytoma is positive for c-kit but usually negative for CK7.^15^

Renin immunohistochemistry staining was negative, ruling out autonomous renin secretion by the tumor cells. Given the tumor's substantial size and extension into the renal sinus, the observed hyperreninemia is most likely secondary to renal hypoperfusion, leading to compensatory activation of the RAAS.

### 3.6 Clinical outcomes and treatment

The clinical outcomes for patients with chRCC are generally favorable, with reported five-year survival rates reaching approximately 85 %.^9^ However, despite the typically good prognosis, certain factors, including large tumor size, vascular invasion, and tumor necrosis, have been associated with poorer outcomes in a minority of cases.^11^

Surgical resection remains the standard treatment for localized chRCC, similar to the approach used for ccRCC.^10^ The significant improvement in the patient's blood pressure following nephrectomy in this case further supports the hypothesis that the tumor was a primary driver of her secondary hypertension.

## 4 Conclusion

This case underscores the importance of considering renal tumors, including rare subtypes such as chRCC, in the differential diagnosis of secondary hypertension, particularly in young patients with resistant hypertension. Early identification of renal masses through imaging and biochemical assessment, followed by timely surgical intervention, remains critical for effective disease management and long-term clinical outcomes.

## CRediT authorship contribution statement

**Tareq Jarrar:** Writing – review & editing, Writing – original draft, Supervision, Methodology, Data curation, Conceptualization. **Younes Jafar Hamam:** Writing – review & editing, Writing – original draft. **Hadeel Abdallah Al Kayed:** Writing – review & editing, Supervision, Methodology, Conceptualization. **Bassel Esmat Alrabadi:** Writing – review & editing, Writing – original draft. **Ayham Kamel Milhem:** Writing – original draft. **Maryam Hisham Alqatawna:** Data curation, Conceptualization. **Husam Odaibat:** Supervision, Conceptualization.

## Informed consent

Written informed consent was obtained from the patient for the publication of this case report and accompanying images.

## Ethics declaration

Written informed consent was obtained from the patient for the publication of this case report and the accompanying images.

## Summary comment

This case highlights the importance of considering renal tumors as a potential cause of secondary hypertension, particularly in young patients with poorly controlled hypertension. It also emphasizes the variability in renal cell carcinoma presentations, where rarer subtypes, such as chRCC, can manifest outside the typical demographic of clear cell RCC. The case underscores the role of biochemical evaluation, imaging, and histopathological assessment in guiding diagnosis and management, reinforcing the necessity for early identification and surgical intervention to achieve optimal patient outcomes.

## Funding declaration

This research did not receive any financial support from public, commercial, or not-for-profit funding agencies.

## Conflict of interest statement

The authors declare no conflicts of interest related to this publication.
